# Surface Reconstruction from Structured Light Images Using Differentiable Rendering

**DOI:** 10.3390/s21041068

**Published:** 2021-02-04

**Authors:** Janus Nørtoft Jensen, Morten Hannemose, J. Andreas Bærentzen, Jakob Wilm, Jeppe Revall Frisvad, Anders Bjorholm Dahl

**Affiliations:** 1 DTU Compute, Technical University of Denmark, 2800 Kgs. Lyngby, Denmark; mohan@dtu.dk (M.H.); janba@dtu.dk (J.A.B.); jerf@dtu.dk (J.R.F.); abda@dtu.dk (A.B.D.); 2 SDU Robotics, University of Southern Denmark, 5230 Odense, Denmark; jaw@mmmi.sdu.dk

**Keywords:** 3D surface reconstruction, 3D scanning, structured light, differentiable rendering

## Abstract

When 3D scanning objects, the objective is usually to obtain a continuous surface. However, most surface scanning methods, such as structured light scanning, yield a point cloud. Obtaining a continuous surface from a point cloud requires a subsequent surface reconstruction step, which is directly affected by any error from the computation of the point cloud. In this work, we propose a one-step approach in which we compute the surface directly from structured light images. Our method minimizes the least-squares error between photographs and renderings of a triangle mesh, where the vertex positions of the mesh are the parameters of the minimization problem. To ensure fast iterations during optimization, we use differentiable rendering, which computes images and gradients in a single pass. We present simulation experiments demonstrating that our method for computing a triangle mesh has several advantages over approaches that rely on an intermediate point cloud. Our method can produce accurate reconstructions when initializing the optimization from a sphere. We also show that our method is good at reconstructing sharp edges and that it is robust with respect to image noise. In addition, our method can improve the output from other reconstruction algorithms if we use these for initialization.

## 1. Introduction

**Figure 1:**
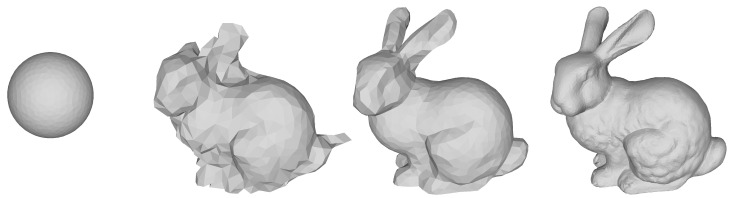
Starting from a sphere, our method reconstructs the Stanford Bunny [3] from images with low levels of noise. From left to right: Initial mesh (sphere), after 50 iterations, after 750 iterations and the converged result (1952 iterations). The final reconstruction has 13,780 vertices and a volume error ($\Delta_{V}$) of 0.30% when compared to the ground truth (depicted in Figure 3j).

Structured light 3D scanning of an object can be used to produce a point cloud from which we can reconstruct a triangle mesh. The resulting mesh is a digital representation of the surface of the scanned object. This has many applications, including cultural heritage preservation and industrial quality control [1,2]. For most applications, the accuracy of the recovered surface of the reconstruction is of great importance. Typically, producing point clouds from phase-shifting structured light images is rather cumbersome. It involves determining the phases, unwrapping these, re-sampling the unwrapped phases due to image distortion and rectification, finding point correspondences and finally triangulating these. Afterwards, the point clouds from different sub-scans need to be merged before the final reconstruction of a triangle mesh. During this process of producing point clouds and subsequently a triangle mesh, the image noise propagates non-linearly to affect vertex positions in the reconstructed triangle mesh. We therefore propose that the point cloud and the process of creating it could be skipped, instead reconstructing surfaces directly from image intensities to investigate how that affects the accuracy of the reconstructions. Using vertex positions as model parameters, we minimize the least-squares error between rendered and recorded images to obtain a triangle mesh directly. An example is presented in Figure 1.

Our method has three major implications: (1) explicit point triangulation from image correspondences is no longer needed; (2) we understand how noise in the image data affects the final reconstruction; and (3) the reconstruction is done for all image data simultaneously.

Similar approaches have been used for multi-view passive stereo but to the best of our knowledge not for structured light reconstructions. In structured light, dense correspondences can be established, usually yielding much higher accuracy in weakly textured areas.

In many reconstruction methods, the raw image data are not considered during the reconstruction [4]. The reconstruction is instead done using unstructured point clouds that have been constructed from the image data. This means that noise due to imaging and point matching processes is not modeled in the reconstruction at all, and this noise is then non-linearly propagated through to the end result. With our approach, the only step in the method in which an error is minimized is in the image intensity domain. This enables us to minimize a more meaningful error, which is especially important in applications with a low signal-to-noise ratio of the reflected light; e.g., when scanning highly specular objects or when using short exposure times for fast acquisition.

## 2. Related Work

Suppose we know the configuration of the light and camera in a vision setup and the reflectance properties of the imaged object. Obtaining the shape of the object based on its shading in an acquired image is then referred to as the shape-from-shading problem [5]. The original shape-from-shading method by Horn [5,6] used so-called characteristic curves to describe the observed shape. The method was based on illumination from point-like sources, and the shading would then only allow the estimation of the gradient along a path. This was the reason for using a collection of curves to describe the object shape.

Curves are inconvenient in the sense that they require stitching to become a full surface description. One way to fit a mesh instead of curves is to use an optimization technique that does not require gradients. This has been done for a rectangular mesh using simulated annealing and simplex search [7]. Gradients are however preferable to ease the optimization problem. For triangle meshes, an approach has been developed based on image gradients [8]. Unfortunately, the shape can be difficult to recover from image gradients due to the color variance caused by normal variations in the surface. To keep gradient-based optimization while developing a method that is more robust to surface reflectance deviating from an assumption of a specific shading model, we use structured light with a differentiable pattern.

The combination of shape from shading with a structured light approach, such as phase-shifting, improves the performance of the shape estimation [9,10] and enables the simultaneous acquisition of shape and object color (diffuse reflectance) [11]. We do not include the estimation of spatially varying reflectance in this study, but we note that this is an option. Only a height field was reconstructed in this previous work. We take this concept one step further and reconstruct a closed 3D object as in the work of Zhang and Seitz [8] but also exploit structured light.

The mesh-based reconstruction method of Zhang and Seitz [8] was improved by Isidoro and Sclaroff [12,13] through the creation of a better initial mesh and by Yu et al. [14,15] using a model that more accurately models the physical reflectance. Another option is to use multiview stereo to acquire a good initial guess and then refine the mesh using a shape-from-shading approach [16]. Our method can be used similarly with the innovation of using structured light to improve the robustness of the mesh refinement.

The use of structured light has become a reliable technique for point-based 3D reconstruction [17] but has, to the best of our knowledge, not previously been tested in mesh-based 3D reconstruction. Our motivation is to have the benefits of a mesh-based technique. An important benefit is that the connectivity between points (vertices) is retained throughout the geometric refinement process.

The recent differentiable renderer from Loubet et al. [18] uses ray tracing and reverse mode automatic differentiation. The use of such a framework for mesh refinement is an option. However, each rendering is quite computationally demanding, so the optimization would have a significant run time. Liu et al. [19] introduced the soft rasterizer, which is a faster differentiable renderer based on a smoothed version of rasterization. This has shown promising results in other mesh reconstruction tasks, but to make it able to render the structured light of a projector is nontrivial.

A different but common approach to reconstructing a shape from structured light images is to first reconstruct a point cloud and then use one of the many methods for the reconstruction of meshes from point clouds [4,20]. Poisson reconstruction [21], which has seen several recent improvements [22], is among the most popular of these. Poisson reconstruction is a volumetric reconstruction method [4]. This means that the reconstruction first finds a smooth characteristic function and then extracts the polygon mesh using a method for iso-contouring [23]. Unfortunately, this means that there is no simple relationship between the final mesh vertices and the points in the intermediate point cloud, making the reconstruction of sharp features challenging, even if the screened variant [24] improved precision. To recapture sharp features, one might apply one of several anisotropic mesh-smoothing methods [25,26,27,28]. While these are effective, they operate solely on the mesh and do not make use of the original images. In particular, this can lead to sharp edges forming without a basis in data. Thus, the point cloud-based pipeline generally does not refer back to the original image data after the point cloud has been reconstructed, meaning that the quantification of the error in the output object is not intrinsic to this pipeline. In comparison, our differentiable rendering-based method optimizes the output mesh based on an energy that directly uses the structured light images.

Another approach is based on the additional information provided by a camera that includes a depth channel (RGB-D). Reconstruction techniques used with depth cameras are, however, either voxel-based or point-based according to a recent survey [29]. Some depth cameras retrieve depth images using structured infrared light. Thus, our technique could be useful for mesh-based reconstruction with a sensor of this kind. Finally, one can rely on the ability of a deep neural network to reconstruct a 3D shape from a single image (or multiple images) [30,31]. In this area, the conclusion is interestingly that a mesh-based method can generate 3D shapes with higher quality than voxel-based and point-based methods. However, while better with a single image, the deep learning-based methods currently lack the universality and adaptability of more traditional methods. Thus, in this work, we suggest a mesh-based method that is not based on deep learning.

## 3. Method

Our method fits a surface to a set of structured light images by minimizing the squared differences between rendered images and real images. We parameterize the object surface by a triangular mesh, and the parameters we optimize are thus the vertex positions v. The real images are captured with structured light phase-shifting from multiple camera-projector positions. We denote these images $I_{c,p}$, where c∈C is the index of the camera–projector pair and p∈P is the index of the projected pattern. We render images ${\tilde{I}}_{c,p}\left(v\right)$ of our parameterized surface from the same camera–projector positions and find the optimal vertex positions by solving the following minimization problem:

(1) $$\underset{v}{\mathrm{argmin}}L\left(v\right)=\underset{v}{\mathrm{argmin}}\sum_{c\in C}\sum_{p\in P}{∥I_{c,p}-{\tilde{I}}_{c,p}\left(v\right)∥}_{F}^{2},$$

where ${∥\cdot ∥}_{F}$ is the Frobenius norm; i.e., we minimize the sum of squared differences over all pixels for all patterns and all camera–projector pairs.

We generate our structured light images by projecting sinusoidal patterns such that the intensity of each column of the projector is given by

(2) $$\frac{1}{2}+\frac{1}{2}\sin\left(2\pi n_{p}x+\phi_{p}\right),$$

where *x* is the *x*-coordinate of the projector normalized to [0,1], $n_{p}$ is the frequency (number of periods) of the pattern and $\phi_{p}$ is a phase-shift. With structured light images, such as phase-shifted images made from Equation (2), the goal is usually to find the *x*-coordinate of the projector, which subsequently can be used for triangulation.

Our method is not specific to phase-shifting patterns; however, we use differentiable patterns to make the minimization problem tractable.

### 3.1. Rendering Images

To solve the minimization problem in Equation (1), we need to render the images ${\tilde{I}}_{c,p}\left(v\right)$ in each iteration. We want to adequately reproduce the structured light images that would have been obtained if our current parameterized surface was a real object. We achieve this by simulating the structured light process as seen from the viewpoint of the camera. We render the images using the formula

(3) $${\tilde{I}}_{c,p}\left(v\right)=A_{c}\sin\left(2\pi n_{p}{\tilde{X}}_{c}\left(v\right)+\phi_{p}\right)+B_{c}.$$

Here, sin(·) is the element-wise application of the sine function, $n_{p}$ is the number of periods as in Equation (2), $\phi_{p}$ is the phase-shift for the *p*th pattern and ${\tilde{X}}_{c}\left(v\right)$ contains the *x*-coordinates of the projector in the [0,1] range for each pixel. We use the *x*-coordinate of the projector as the projector is offset from the camera along its *x*-axis. The matrices $A_{c}$ and $B_{c}$ are amplitudes and biases that are estimated from the ground truth images by fitting sinusoids at each pixel location. We find the elements of ${\tilde{X}}_{c}\left(v\right)$ by tracing a ray from the camera through the center of each pixel and projecting the point where it intersects the surface back to the projector. As we model the projector as a pinhole camera, the point is projected to the projector as follows:

(4) $$\left[\begin{array}{c} q_{1}\\ q_{2}\\ q_{3} \end{array}\right]=P\left[\begin{array}{c} r\\ 1 \end{array}\right]=\left[\begin{array}{cccc} p_{1} & p_{2} & p_{3} & p_{4} \end{array}\right]\left[\begin{array}{c} r\\ 1 \end{array}\right],$$

where P is the projection matrix of the projector, $p_{i}$ is the *i*th column of P and r is the 3D point where the ray intersects the triangle face. The i,jth element of ${\tilde{X}}_{c}$ is then given by

(5) $${\tilde{X}}_{c}^{i,j}=\frac{q_{1}}{q_{3}}.$$

If the ray does not intersect the surface, we treat the pixel as background and set ${\tilde{X}}_{c}^{i,j}\left(v\right):=X_{c}^{i,j}$ such that the corresponding term in the loss L(v) will be zero.

Note that $2A_{c}$ is the proportion of projector light that is reflected into the camera and $B_{c}-A_{c}$ is the amount of global light. We estimate these to allow the renderings to resemble the true images better. Fortunately, we need only estimate them once for each viewpoint, as we can then use these estimates repeatedly in each iteration of the optimization.

### 3.2. Optimizing the Surface

We use gradient descent to solve the optimization problem in Equation (1). In order to do this, we need the gradient of L(v), which in turn depends on the gradient of the elements in ${\tilde{X}}_{c}\left(v\right)$. Recall that each of these elements is computed by tracing a single ray from the camera to the surface of the object. The gradient of ${\tilde{X}}_{c}^{i,j}$ will therefore only have contributions from the vertices spanning the triangle face that intersects the ray. We can compute the derivative for one of these three vertices ($p_{a}$) as follows:

(6) $$\begin{array}{cc} \frac{\partial {\tilde{X}}_{c}^{i,j}}{\partial p_{a}}=\left(\frac{p_{1}-{\tilde{X}}_{c}^{i,j}p_{3}}{q_{3}}\cdot d\right) & \frac{\lambda_{a}n}{d\cdot n}, \end{array}$$

where d is the ray direction, n is the normal of the face, and $\lambda_{a}$ is the barycentric coordinate corresponding to $p_{a}$. The equations for the remaining two vertices, $p_{b}$ and $p_{c}$, use $\lambda_{b}$ and $\lambda_{c}$ but are otherwise identical. For a derivation of Equation (6), see Appendix A.

As mentioned, we use gradient descent to update the vertex positions; that is,

(7) $$v_{i+1}=v_{i}-\alpha_{i}\nabla L\left(v_{i}\right),$$

where $v_{i}$ and $v_{i+1}$ are the vertex positions in the *i*th and (i+1)th iterations, respectively. To choose the step-length $\alpha_{i}$, we use a simple backtracking line-search [32] to choose

(8) $$\alpha_{i}=\frac{1}{2^{n}}\alpha ,$$

where α is a fixed constant and *n* is the smallest non-negative integer such that $L\left(v_{i+1}\right)<L\left(v_{i}\right)$ when doing the update.

### 3.3. Initializing the Optimization

We have so far described how the iterative part of the optimization problem works, but this is only one half of the problem. A good initial guess is extremely important to ensure convergence. In the next two sections, we introduce two possible ways to obtain an initial guess for the minimization problem in Equation (1).

#### 3.3.1. Using Other Reconstruction Methods

One way of getting a good initial guess is by using a reconstruction found via another reconstruction method. In this way, our method can be seen as a post-processing step that tries to adjust the reconstruction to fit to the original image data better. In some of our experiments, we have used Screened Poisson Reconstructions [24] at various depths as initialization. This method is used as it is able to produce decent reconstructions even from a very noisy point cloud and therefore yields a good initial guess. However, as Poisson reconstructions often produce undesired triangulations, we have found it to be beneficial to remesh the mesh before starting the optimization.

#### 3.3.2. Using a Simple Shape

Another way of obtaining an initial guess is to use a simple shape such as a sphere. However, as the objective in Equation (1) has many high-frequency sinusoids, the optimization is prone to ending up in local minima when the initial guess is far from a global minimum. We therefore propose solving a related, but simpler, minimization problem and to use the solution as an initial guess for our problem in Equation (1). The simpler problem is given by

(9) $$\underset{v}{\mathrm{argmin}}\sum_{c\in C}{∥X_{c}-{\tilde{X}}_{c}\left(v\right)∥}_{F}^{2},$$

where $X_{c}$ denotes the *x*-coordinates of the projector from the ground truth images. The method used to recover $X_{c}$ depends on the patterns displayed, but for two sets of phase-shifted patterns, the heterodyne principle can be used [33].

To make the initial problem simpler, we start with a mesh that has few vertices and gradually increase the number of vertices by remeshing. This enables us to use a simple shape, e.g., a sphere, as our initial mesh.

#### 3.3.3. Remeshing

As described in Section 3.3.1 and Section 3.3.2, we use a remeshing algorithm. The algorithm we use is adapted from the Python geometry processing library Pymesh [34]. The first step is to remove any degenerate triangles and to split all edges that are longer than *ℓ*. Then, the algorithm repeatedly collapses any edges that are shorter than *ℓ* and splits any obtuse triangles where the angle is greater than 150°. This collapsing and splitting is repeated until the mesh no longer changes. Finally, any self-intersections are removed, the mesh is replaced by the outer hull of the mesh, obtuse triangles with an angle above 179° are split and any isolated vertices are removed.

## 4. Experiments

**Figure 2:**
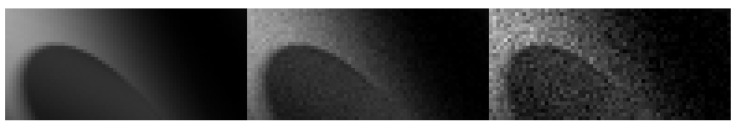
Crop of image shown with varying levels of noise. From left to right: k=1, $k=10^{2}$, $k=10^{3}$.

To demonstrate the usefulness of our method, we carried out a few experiments that we briefly introduce here. First, we showed that our method can reconstruct an object starting from a sphere; see Figure 1. Secondly, we reconstructed three different objects at multiple levels of noise; see Figure 2. Finally, we compared our method against a Poisson reconstruction at two levels of noise and showed that our method could reconstruct sharp edges.

### 4.1. Generating Ground Truth Images

We performed all experiments using synthetic ground truth images in order to have access to the ground truth shape of the mesh for comparison. Our ground truth images were made by projecting two sets of phase-shifted patterns with fifteen and sixteen periods, respectively, with sixteen shifts of the first pattern and eight shifts of the second, such that

(10) $$n_{p}=\left\{\begin{array}{cc} 15 & p\in [1,2,\cdots ,16]\\ 16 & p\in [17,18,\cdots ,24] \end{array}\right.$$

and

(11) $$\phi_{p}=\left\{\begin{array}{cc} 2\pi p\frac{1}{16} & p\in [1,2,\cdots ,16]\\ 2\pi \left(p-16\right)\frac{1}{8} & p\in [17,18,\cdots ,24]. \end{array}\right.$$

#### 4.1.1. Rendering

We rendered the ground truth images using ray tracing with 100 samples per pixel for anti-aliasing, and we used the Lambertian reflectance model to describe the optical properties of our objects of interest.

#### 4.1.2. Noise

As these ground truth images were noise-free, we added noise to make the images more realistic. For this, we modeled the noise of a pixel with intensity *x* by a Gaussian distribution with mean *x* and the following variance:

(12) $$\sigma^{2}=\sigma_{r}^{2}+x\sigma_{p}^{2},$$

where the first term $\sigma_{r}$ describes the signal-independent sensor read-out noise and the second term $x\sigma_{p}$ describes the signal-dependent shot noise. We chose $\sigma_{r}$ and $\sigma_{p}$ by using the noise levels from a baseline camera [35]. Our noise levels were then defined as multiples of this baseline noise level, controlled by *k* as follows:

(13) $$\sigma^{2}\left(x,k\right)=k\left(4.5\cdot 10^{-7}+x\cdot 2\cdot 10^{-5}\right),$$

such that k=1 gives the noise levels of a baseline camera for x∈[0,1]. After adding noise, we clamped pixel values to the [0,1] range. Examples of images for different values of *k* are presented in Figure 2.

### 4.2. Quantitative Evaluation

We quantitatively evaluated the performance of our method using a metric $\Delta_{V}$ closely related to one minus the volumetric intersection over union (IoU):

(14) $$\Delta_{V}=\frac{\left|\left(S\backslash \tilde{S}\right)\cup \left(\tilde{S}\backslash S\right)\right|}{\left|S\right|},$$

where *S* and $\tilde{S}$ are the ground truth and reconstruction considered as solids, and |·| is the volume of a solid. Volumetric IoU has recently been used for 3D object detection [36] and for comparing volume-based surface representations [37,38,39,40]. As demonstrated by Kato et al. [40], the metric is equally useful for a mesh-based surface representation like the one we use.

### 4.3. Experiment Details

**Figure 3:**
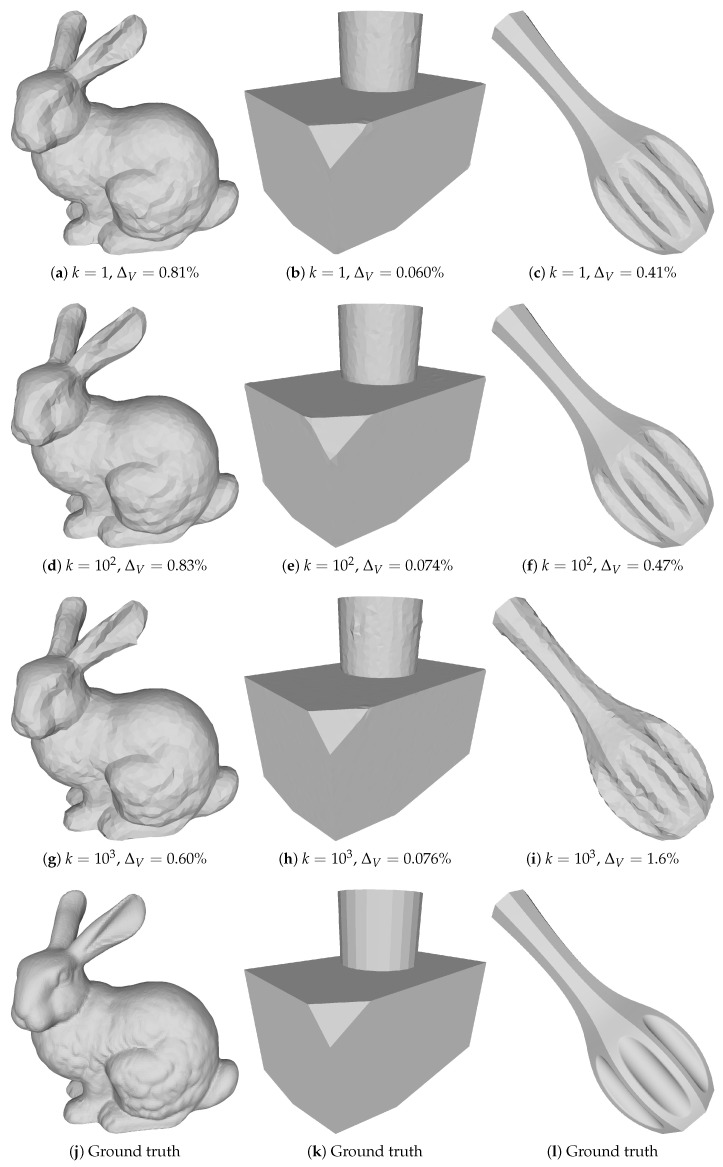
Reconstructions made with our method on three different objects, for increasing levels of noise *k*.

**Figure 4:**
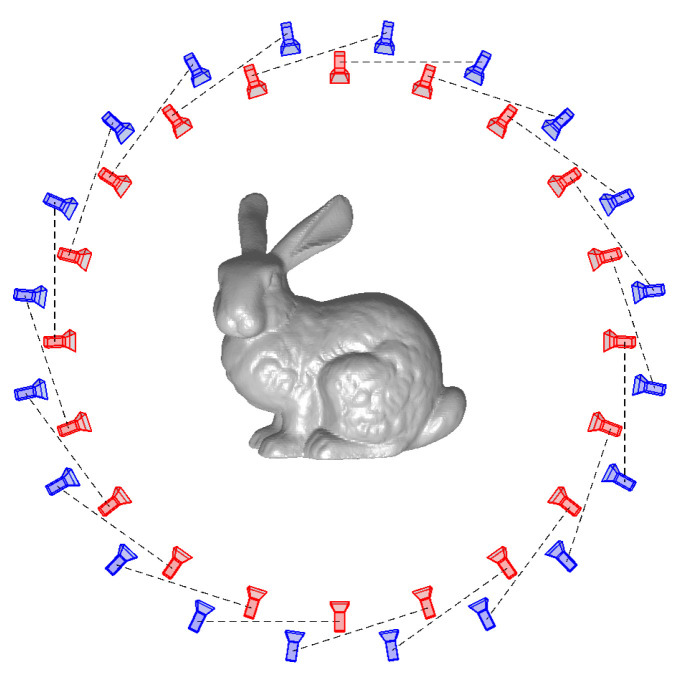
Visualization of one of the circles from our camera–projector setup. In all experiments, we used three circles with 20 cameras each. Red indicates a camera and blue indicates a projector. The dotted lines show which cameras and projectors belong together.

We evaluated our method on three different shapes: the Stanford Bunny [3], a combination of a cylinder and a box with various truncated corners and a dandelion vase [41]. These shapes are presented in the bottom row of Figure 3. When starting from a simple shape as described in Section 3.3.2, we used the same sphere across all our experiments. When optimizing the simpler problem in Equation (9), we performed remeshing for every 25th iteration. At each remeshing step, we decreased the target edge length, which yielded meshes with an increasingly fine resolution. For the *i*th remeshing step, we set the target to

(15) $$\ell_{i}=0.99^{i}\cdot 0.025\cdot d_{BB},$$

where $d_{BB}$ is the largest diagonal of the bounding box of the current mesh. To be able to solve the problem in Equation (9), we estimated $X_{c}$ using the heterodyne principle [33]. In all our experiments, we used 60 camera–projector positions organized in three circles, with 20 cameras in each circle, to mimic a structured light scanner with the object placed in three different poses on a turntable rotating 18° between each image. One of these circles is visualized in Figure 4. The camera resolution for all renderings was 1920×1080 pixels. Our method used approximately 1 s per iteration depending on the number of vertices and the resolution of the images.

In Figure 1, we show how our method was able to reconstruct the Stanford Bunny starting from a sphere. The reconstruction was done for k=1. After finishing the optimization based on Equation (9), we remeshed the result by halving the target edge length *ℓ* from the last remeshing step to get a mesh with an even finer resolution. The two in-progress images shown are during the initial optimization directly on $X_{c}$. The final reconstruction contained many of the fine details of the true bunny (Figure 3j).

**Figure 5:**
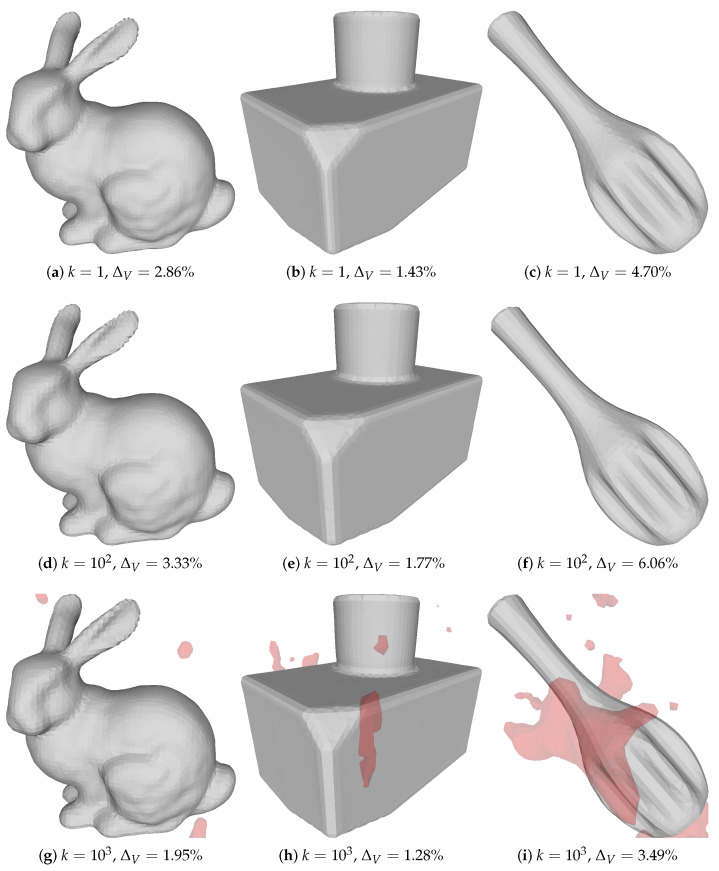
Poisson reconstructions of three different objects, for increasing levels of noise *k*. The reconstructions for $k=10^{3}$ had a lower $\Delta_{V}$ than the reconstructions at lower noise levels, as these reconstructions were done at an increased octree depth to ensure that the reconstructions had more vertices than the corresponding reconstructions in Figure 3. Red areas do not belong to the largest connected component and have thus not been used in the computation of $\Delta_{V}$.

To examine how our method is affected by noise, we reconstructed all three objects starting from a sphere with varying levels of image noise ($k\in [1,10^{2},10^{3}]$), which we show in Figure 3. We see that our method was largely unaffected by noise and was still able to reconstruct the shape. Figure 5 shows the corresponding results when using a screened Poisson reconstruction.

**Figure 6:**
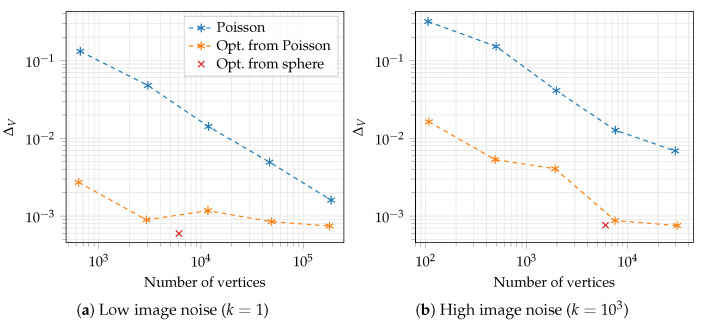
Comparison showing $\Delta_{V}$ as a function of the number of vertices in the mesh for reconstructions of a box with a cylinder. Poisson is the Poisson reconstruction, where only the connected component with the largest volume has been kept, as the Poisson reconstruction sometimes produces multiple connected components for high levels of noise. Opt. from Poisson and from sphere presents our method using the initial guesses described in Section 3.3. The Poisson reconstruction was done for spatial octree depths of 4 to 8.

**Figure 7:**
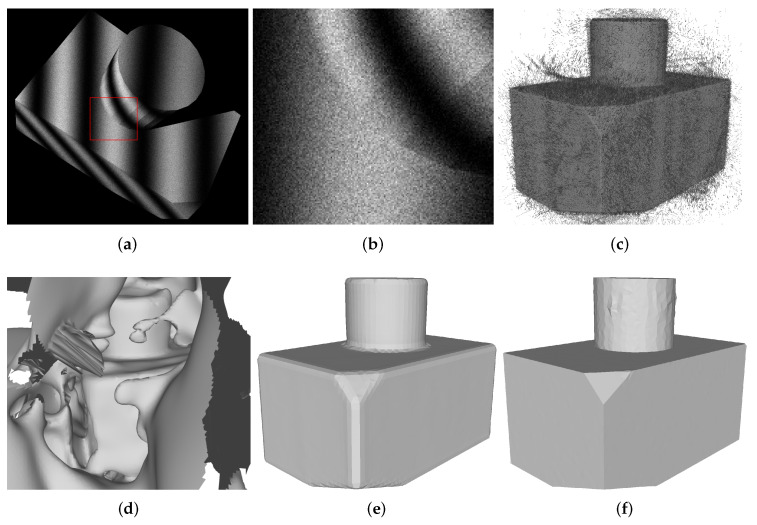
Our method was able to reconstruct meshes with sharp edges from very noisy images ($k=10^{3}$). Our method had a lower error ($\Delta_{V}$) than a Screened Poisson reconstruction with a comparable number of vertices (spatial octree depth 7). The error of the Robust Implicit Moving Least Squares (RIMLS) reconstruction could not be evaluated. (**a**) A noisy input image. (**b**) Crop of noisy image. (**c**) Point cloud. (**d**) RIMLS reconstruction [42]. (**e**) Poisson reconstruction [24] with 7064 vertices. $\Delta_{V}=1.28\%$. (**f**) our method with 6038 vertices. $\Delta_{V}=0.08\%$.

Finally, we compared our method against Screened Poisson reconstruction, as shown in Figure 6, for multiple depths of the reconstruction. The point cloud used for the Poisson reconstruction for k=1 contained 22 million points, and the point cloud for $k=10^{3}$ contained 17 million points. Our method consistently achieved a lower error than the Poisson reconstruction. The meshes from some of these data points are shown in Figure 7 along with a Robust Implicit Moving Least Squares (RIMLS) reconstruction. Figure 7 also shows that our method was able to reconstruct sharp features even in the presence of large amounts of noise compared to other feature-preserving reconstruction methods such as RIMLS [42]. The result from RIMLS was very poor in this case due to the large amount of noise in the point cloud.

## 5. Discussion

Our method has the same implicit assumption about the appearance of the object that is necessary for structured light. This assumption is that the amount of global light in each pixel, i.e., the light that is not directly reflected, is the same for all patterns. This is approximately true for high-frequency sinusoidal patterns [43].

Although we used the Lambertian reflectance model to render our synthetic data, we do not expect this to be a limitation of our method. It does not rely on the assumption of Lambertian reflectance due to the use of structured light, and therefore does not rely on estimating a texture map of the object, neither implicitly nor explicitly.

In our experiments, we had the advantage of knowing the camera and projector positions exactly, which would not be the case when working with real data. However, this can be remedied by including the positions of these as parameters in the optimization problem. Implementing this is relatively easy as our method only requires first-order derivatives, which can be computed analytically using an approach similar to that used in Appendix A. Additionally, we do not need to purely rely on our estimates of $A_{c}$, $B_{c}$, as these can also be part of the optimized parameters. However, we expect both $A_{c}$, $B_{c}$, and the camera–projector positions to be known quite accurately and would suggest allowing them to be part of the optimization only once the original optimization problem has converged.

In order to solve the optimization problem, we computed the derivative of our loss function, which involved the derivative of our rendering. This is potentially problematic as our rendering is not differentiable at depth discontinuities; i.e., where non-neighbouring faces of the mesh are bordering each other in the image space. This could, e.g., be the ear and body of the Stanford Bunny. Our method will in this case experience aliasing error in the renderings and derivatives. However, since the faces are observed from multiple views simultaneously, there is often another view in which the same edges are observed with continuous depth. The problem is thus mitigated, and since it occurs only for a small percentage of the pixels for each iteration, it was not a problem in our optimization experiments. Although the optimization problem in Equation (9) is much less prone to ending up in a local minimum, it is still not guaranteed to find the global optimum. However, as the graphs in Figure 6 demonstrate, our method is able to find a good local minimum.

The choice of using a mesh as the surface representation to optimize has some advantages. It is very efficient to compute the gradient of our loss function for a mesh, as each pixel only influences a constant number of elements in the gradient, which makes it suitable for parallel implementation on a GPU (Graphics Processing Unit).

### Limitations

A disadvantage of our method is that it is not able to handle topology changes. In practice, this means that the initial mesh must have the same topology as the true object, but even then it is possible that the initial shape would need to be closer to the final shape for objects containing holes. Whether starting with a simple shape with the correct topology is sufficient is yet to be determined. However, this problem is avoided when using a Poisson reconstruction as the initial guess.

While our method is quite robust to image noise, there will be extreme situations where the structured light decoding scheme will fail in so many pixels that either of our initialization methods will fail. This is because both of our initial guesses to the method rely on having access to ${\tilde{X}}_{c}$, which in the extreme case only contains noise. The limit to how much noise we can handle is yet to be determined, but as seen in Figure 2 and Figure 3, we are able to handle substantial amounts of image noise.

We can control the complexity of our mesh by adjusting the target number of vertices. This is a feature and in some cases also a limitation. The maximum target number of vertices is bounded by the amount of image noise. When the target number of vertices is increased, each face in the mesh becomes smaller and therefore is more affected by noise. Being able to set the number of vertices in the mesh, however, also allows the user to determine how many vertices are required to describe the given geometry. The number of vertices also implicitly controls the amount of smoothing our method does, which enables control over the trade-off between bias and variance.

Areas of the object that have not been observed by any of the cameras are not affected by any gradient updates and therefore retain the shape of the initial guess. This means that full coverage of the object is necessary to obtain an accurate reconstruction. However, our method is naturally able to identify which areas are not seen by any camera in the final solution, which implies that our method can be extended to remove or smooth these areas depending on the requirements.

## 6. Conclusions

Our primary contribution in this work is a novel model for computing a surface mesh directly from structured light images without the need for an intermediate point cloud. With this model, we have shown that the direct computation of a triangle mesh gives high accuracy and is particularly good at reconstructing sharp features such as corners and edges, which are smoothed out using the two-step Screened Poisson reconstruction that we used for comparison. Further, we have obtained very high robustness to noise by optimizing the vertex positions directly from the images. Finally, our approach completely avoids partial scans that must subsequently be aligned, because the optimization is done for all images at once. This demonstrates the advantage of directly reconstructing a surface from structured light images using differentiable rendering. Our method opens up many interesting avenues of future work, including bringing it from the realm of synthetic data to real-world data and testing the limits with respect to image noise. We conjecture that our method may be well-suited for scanning highly specular objects and other objects with a low signal-to-noise-ratio where aggregating information from many views is beneficial.

## Data Availability

The data used in this study are available at https://eco3d.compute.dtu.dk/pages/scanning.

## Appendix A. Derivation of Equation (6)

In this appendix, we derive the gradients of the elements of ${\tilde{X}}_{c}\left(v\right)$ with respect to the vertices v. Let ${\tilde{X}}_{c}^{i,j}$ be the i,jth element of ${\tilde{X}}_{c}\left(v\right).$ This is found by tracing a ray through the center of the i,jth pixel in the camera until it intersects the surface at a point r; that is,

(A1) r=o+td,

for some *t*, where o is the position of the camera and d is the direction of the ray. Modeling the projector as a pinhole camera, this point is projected back into the projector by

(A2) $$\begin{array}{cc} \left[\begin{array}{c} q_{1}\\ q_{2}\\ q_{3} \end{array}\right] & =\left[\begin{array}{cccc} P_{1,1} & P_{1,2} & P_{1,3} & P_{1,4}\\ P_{2,1} & P_{2,2} & P_{2,3} & P_{2,4}\\ P_{3,1} & P_{3,2} & P_{3,3} & P_{3,4} \end{array}\right]\left[\begin{array}{c} r\\ 1 \end{array}\right]. \end{array}$$

If we define $p_{1}=\left[\begin{array}{ccc} P_{1,1} & P_{1,2} & P_{1,3} \end{array}\right]$ and $p_{3}=\left[\begin{array}{ccc} P_{3,1} & P_{3,2} & P_{3,3} \end{array}\right]$ then,

(A3) $${\tilde{X}}_{c}^{i,j}=\frac{q_{1}}{q_{3}}=\frac{p_{1}\cdot r+P_{1,4}}{p_{3}\cdot r+P_{3,4}}.$$

The point r intersects a triangle with vertices $p_{a},p_{b},$ and $p_{c}$. The normal of this triangle can be computed by

(A4) $$n=\left(p_{c}-p_{b}\right)\times \left(p_{a}-p_{c}\right).$$

Using the normal, we can write *t* as

(A5) $$t=\frac{(p_{b}-o)\cdot n}{d\cdot n}.$$

Using the chain-rule, we obtain that

(A6) $$\begin{array}{cc} \frac{\partial {\tilde{X}}_{c}^{i,j}}{\partial p_{a}} & =\frac{\partial {\tilde{X}}_{c}^{i,j}}{\partial t}\frac{\partial t}{\partial p_{a}} \end{array}$$

(A7) $$\begin{array}{cc} & =\left(\sum_{i=1}^{3}\frac{\partial {\tilde{X}}_{c}^{i,j}}{\partial p_{i}}\frac{\partial p_{i}}{\partial t}\right)\frac{\partial t}{\partial p_{a}} \end{array}$$

(A8) $$\begin{array}{cc} & =\left(\frac{\partial {\tilde{X}}_{c}^{i,j}}{\partial r}\cdot d\right)\frac{\partial t}{\partial p_{a}}. \end{array}$$

Here,

(A9) $$\begin{array}{cc} \frac{\partial {\tilde{X}}_{c}^{i,j}}{\partial r} & =\frac{\frac{\partial q_{1}}{\partial r}q_{3}-q_{1}\frac{\partial q_{3}}{\partial r}}{{q_{3}}^{2}} \end{array}$$

(A10) $$\begin{array}{cc} & =\frac{\frac{\partial q_{1}}{\partial r}-{\tilde{X}}_{c}^{i,j}\frac{\partial q_{3}}{\partial r}}{q_{3}} \end{array}$$

(A11) $$\begin{array}{cc} & =\frac{p_{1}-{\tilde{X}}_{c}^{i,j}p_{3}}{q_{3}}. \end{array}$$

The last missing term in Equation (A8) is

(A12) $$\begin{array}{cc} \frac{\partial t}{\partial p_{a}} & =\frac{\partial }{\partial p_{a}}\frac{(p_{b}-o)\cdot n}{d\cdot n} \end{array}$$

(A13) $$\begin{array}{cc} & =\frac{\frac{\partial }{\partial p_{a}}\left[(p_{b}-o)\cdot n\right]\left(d\cdot n\right)-\left[(p_{b}-o)\cdot n\right]\frac{\partial d\cdot n}{\partial p_{a}}}{{\left(d\cdot n\right)}^{2}} \end{array}$$

(A14) $$\begin{array}{cc} & =\frac{{\frac{\partial n}{\partial p_{a}}}^{⊤}\left(p_{b}-o\right)-t{\frac{\partial n}{\partial p_{a}}}^{⊤}d}{d\cdot n} \end{array}$$

(A15) $$\begin{array}{cc} & =\frac{{\frac{\partial n}{\partial p_{a}}}^{⊤}\left(p_{b}-o-td\right)}{d\cdot n} \end{array}$$

(A16) $$\begin{array}{cc} & =\frac{{\frac{\partial n}{\partial p_{a}}}^{⊤}\left(p_{b}-r\right)}{d\cdot n}, \end{array}$$

with

(A17) $$\begin{array}{cc} \frac{\partial n}{\partial p_{a}} & =\frac{\partial }{\partial p_{a}}\left(p_{c}-p_{b}\right)\times \left(p_{a}-p_{c}\right) \end{array}$$

(A18) $$\begin{array}{cc} & =\frac{\partial }{\partial p_{a}}\left(p_{c}-p_{b}\right)\times p_{a} \end{array}$$

(A19) $$\begin{array}{cc} & =\frac{\partial }{\partial p_{a}}{\left[p_{c}-p_{b}\right]}_{\times }p_{a} \end{array}$$

(A20) $$\begin{array}{cc} & ={\left[p_{c}-p_{b}\right]}_{\times }, \end{array}$$

where we utilize that a cross-product a×b can be written as a matrix-vector product ${\left[a\right]}_{\times }b$ using a skew-symmetric matrix. Inserting this into Equation (A16), we get

(A21) $$\begin{array}{cc} \frac{\partial t}{\partial p_{a}} & =\frac{\left(p_{b}-r\right)\times \left(p_{c}-p_{b}\right)}{d\cdot n}. \end{array}$$

If we write r using barycentric coordinates, $r=\lambda_{a}p_{a}+\lambda_{b}p_{b}+\lambda_{c}p_{c}$ with $\lambda_{a}+\lambda_{b}+\lambda_{c}=1$, then Equation (A21) reduces to

(A22) $$\begin{array}{cc} \frac{\partial t}{\partial p_{a}} & =\frac{\left(p_{b}-\left(\lambda_{a}p_{a}+\lambda_{b}p_{b}+\lambda_{c}p_{c}\right)\right)\times \left(p_{c}-p_{b}\right)}{d\cdot n}\\ & =\frac{\left(\lambda_{a}\left(p_{c}-p_{a}\right)+\left(1-\lambda_{b}\right)\left(p_{b}-p_{c}\right)\right)\times \left(p_{c}-p_{b}\right)}{d\cdot n}\\ & =\frac{\lambda_{a}\left(p_{c}-p_{a}\right)\times \left(p_{c}-p_{b}\right)}{d\cdot n}\\ & =\frac{\lambda_{a}n}{d\cdot n}. \end{array}$$

Finally, we obtain (Equation (6))

(A23) $$\begin{array}{cc} \frac{\partial {\tilde{X}}_{c}^{i,j}}{\partial p_{a}}=\left(\frac{p_{1}-{\tilde{X}}_{c}^{i,j}p_{3}}{q_{3}}\cdot d\right) & \frac{\lambda_{a}n}{d\cdot n}. \end{array}$$

The derivatives with respect to $p_{b}$, and $p_{c}$ can be found with similar derivations.
